# Nanoindentation Analysis of SU-8 Coated Wafers at Different Baking Phases

**DOI:** 10.3390/polym17243337

**Published:** 2025-12-18

**Authors:** Tamás Tarjányi, Gábor Gulyás, Krisztián Bali, Márton Sámi, Rebeka Anna Kiss, Barbara Beiler, Péter Fürjes, Tibor Szabó

**Affiliations:** 1 Department of Medical Physics and Informatics, University of Szeged, 6720 Szeged, Hungary; kissrebeka0125@gmail.com (R.A.K.); tiberatosz@gmail.com (T.S.); 2 Department of Optics and Quantum Electronics, University of Szeged, 6720 Szeged, Hungary; gulyasg@titan.physx.u-szeged.hu (G.G.); samimarton11@gmail.com (M.S.); 3 SEMILAB Semiconductor Physics Laboratory Co., Ltd., 1117 Budapest, Hungary; krisztian.bali@semilab.hu; 4 Institute of Technical Physics and Materials Science, HUN-REN Centre for Energy Research, Konkoly-Thege Miklós Str. 29-33, 1121 Budapest, Hungary; beiler.barbara@ek.hun-ren.hu (B.B.); furjes.peter@ek.hun-ren.hu (P.F.)

**Keywords:** nanoindentation, hardness, creep, wafer, SU-8, epoxy

## Abstract

SU-8 photoresist is extensively used as a structural and passivation layer in microelectromechanical systems, microfluidic devices, and related microscale technologies. The long-term reliability of these devices critically depends on the mechanical integrity and viscoelastic behaviour of the SU-8 coating. In this study, the mechanical and viscoelastic behaviour of SU-8 polymer thin films was systematically investigated using nanoindentation at different baking stages representative of standard photolithographic processing. SU-8 layers were spin-coated on silicon wafers and subjected to pre-bake, post-bake, and hard-bake treatments to evaluate the effects of progressive cross-linking. Static nanoindentation revealed that the elastic modulus did not change significantly during the baking phases and remained near 6.2 GPa; however, a significant change in hardness was observed from 0.173 ± 0.012 GPa after pre-bake to 0.365 ± 0.011 GPa and 0.364 ± 0.016 GPa after post- and hard bake, respectively. Creep tests analysed by the Burgers viscoelastic model showed a significant increase in both the retarded modulus and viscosity parameters with thermal curing, indicating the suppression of long-term viscoelastic deformation. The combined results demonstrate that nanoindentation provides a sensitive, nondestructive tool for monitoring the evolution of cross-linking and viscoelastic stability in SU-8 films, offering valuable insight for process optimization and mechanical reliability in MEMS and microfluidic applications.

## 1. Introduction

The development of micro- and nanofabrication techniques has received interest in terms of the mechanical properties of photocurable polymers, such as SU-8, which can be used in the case of silicon micromachining [1,2,3]. These photoresist layers play a critical role in defining the structures of microelectromechanical systems (MEMS), biomedical microdevices, or integrated microsystems [4]. SU-8 is a negative-tone, epoxy-based photoresist commonly used in MEMS and bioMEMS devices. It is usually applied as a thin film on silicon or glass wafer substrates and then exposed to ultraviolet light through a mask, creating precise patterns [4,5]. After curing, these systems show excellent chemical stability, mechanical strength, and ability to form thick, high-aspect-ratio structures [6,7,8]. Its versatility allows it to be used in applications such as sensors, microfluidics, or optical devices. The mechanical integrity of SU-8 layers is crucial for device performance and long-term reliability, particularly when they serve as structural or encapsulating components exposed to mechanical or thermal loading. Understanding their mechanical properties during manufacturing phases can also be beneficial in optimizing the fabrication process, enhancing the reliability of electronic devices.

The raw SU-8 material contains an epoxy-based monomer (Bisphenol A Novolac) and photocatalytic initiator (Triarylsulfonium/hexafluoroantimonate salt) dissolved in an organic solvent (gamma-butyrolactone or cyclopentanone) [9]. Due to UV exposure, the generated photoacid (hexafluoroantimonic) promotes the thermally activated reactions of the epoxy monomers through their neutral epoxy groups to form a cross-linked polymer network [5]. By varying the curing parameters (UV dose and exposure time, baking temperature, and cycle times), the mechanical properties of the film can be controlled. To optimize the thermal process of the SU-8 layer, a typical baking program consists of multiple steps using room temperature cycles—65 –95 °C—in both pre-bake (or soft bake) and post-bake (post-exposure bake—PEB) cases. The completely cross-linked resist is characterized by excellent mechanical properties, although to improve its hardness, a further hard-bake process can be considered [10].

The polymer network of SU-8 evolves through solvent evaporation and cross-linking during the sequential UV exposure and baking steps of lithographic processes, and its mechanical response is highly dependent on the degree of curing and the resulting cross-link density. The lifespan of the final SU-80based products is significantly affected by the hardness, stiffness, or viscoelastic behaviour of the material [11].

The fabrication of SU-8 structures on the silicon wafers involves several thermal treatments, namely pre-bake (often called soft bake), post-bake (or post-exposure bake implemented after lithography, the UV exposure in this context), and hard bake [10,12]. These baking steps affect mechanical and chemical properties due to the induced changes in the polymer structure and the density of the cross-linking. The pre-bake step is described to primarily remove solvents and enhance adhesion to the substrate. This phase significantly affects the mechanical stability of the resist. The post-bake step initiates extensive cross-linking reactions and can greatly impact the hardness of the surface layer. The last baking phase, hard bake, is usually an optional step and is used to stabilize the SU-8 polymer further to remove residual mechanical stresses, improving long-term stability [13,14]. Most previous investigations examined either static mechanical properties or high-temperature viscoelasticity, while few studies have correlated the evolution of viscoelastic parameters with the specific baking stages used in microfabrication.

A modern approach to testing the mechanical properties is the nanoindentation method. Nanoindentation is described as a sensitive non-destructive method and can be used to characterize properties of thin films and nanostructured or even bulk materials [11,15]. It can give a reproducible, detailed picture of the hardness, elastic modulus, creep, or dynamic behaviour of samples at a high spatial resolution. To test silicon wafers or MEMS properties, such high-precision devices are well-suited. The nanoindenter tools, in general, apply a load through a sharp indenter tip, and the force and displacement are measured continuously. The obtained load–displacement curves can be analysed to investigate the hardness or modulus of elasticity of the material through the Oliver–Pharr method [15].

Beyond conventional microfabrication polymers, recent studies on polymer-based thin films have highlighted that mechanical integrity, stress transfer efficiency, and stiffness evolution critically govern device reliability and functional stability in advanced flexible and microstructured systems [16,17]. In such materials, quantification of elastic parameters and time-dependent mechanical properties is essential for understanding structure–property relationships for optimizing processing conditions [18].

The present study aims to fill this gap by providing a systematic nanoindentation-based assessment of both the instantaneous and time-dependent mechanical behaviour of SU-8 coatings on silicon wafer under different baking conditions that are directly relevant to lithographic fabrication. Static indentation data were complemented by creep experiments analysed using the Burgers viscoelastic model to quantify the changes in the elastic moduli and viscosity parameters associated with progressive cross-linking. By comparing these results with established literature data, we elucidate how processing temperature governs the transition from viscous to viscoelastic–elastic behaviour and propose nanoindentation as a rapid, nondestructive tool for monitoring polymerization and mechanical stability in SU-8-based microfabrication.

## 2. Materials and Methods

### 2.1. Sample Preparation

**Figure 1:**
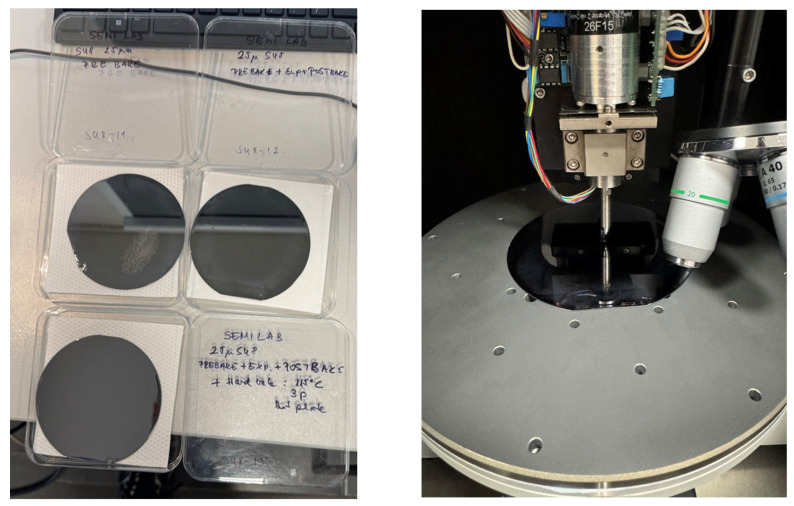
The finished SU-8-coated silicon wafer samples from different baking steps are shown on the left, and the nanoindenter instrument used for mechanical measurements is shown on the right, equipped with a diamond Berkovich tip. The wafer samples were fixed with a vacuum chuck during measurements.

Single-crystal silicon wafer substrate samples were chemically cleaned in a nitric acid bath at 90 °C to remove surface contaminants, washed in DI water, dried by N_2_, and dehydrated at 300 °C for 30 min on a hotplate to eliminate residual moisture. The surface of the samples was coated with SU-8 2025-type photoresist using the spin-coating technique at 3000 rpm for 30 s to achieve uniform thickness (22–25 μm). The first sample was only pre-baked on a hotplate (Brewer Science CEE 200CBX spin-bake and CEE 1300X precision bake system, Cost Effective Equipment, St. James, MO, USA), applying the conventional temperature sequence of 65 °C for 1 min, 95 °C for 5 min, and room temperature for 10 min. The wafer samples were placed directly onto the preheated hotplates in sequence. After pre-bake, the second sample was exposed to UV light (using SUSS MicroTec MA6 mask aligner system with UV400 broad (250–450 nm), SUSS MicroTec, Garching, Germany) wavelength range at 250 mJ/cm^2^ exposure energy) and post-baked with the same thermal cycle described above. The third sample was hard-baked at 115 °C for 3 min as the final treatment after the pre-bake–exposure–post-bake sequence (see Figure 1).

### 2.2. Static Nanoindentation Protocol

**Figure 2:**
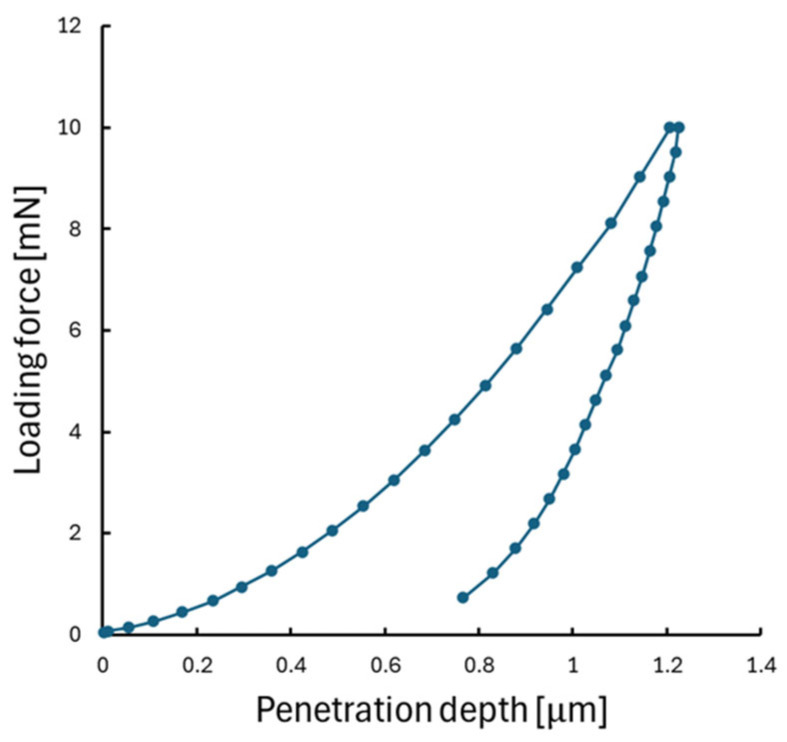
A representative force–displacement curve obtained from the static nanoindentation measurement on the pre-baked SU-8-coated silicon wafer. The curve illustrates a 10 mN loading and a short 1 s holding and unloading phase during the static test.

The Semilab IND-1500 nanoindenter tool (Semilab, Budapest, Hungary) was used for nanoindentation measurements. The SU-8-coated silicon wafer samples were fixed with a vacuum chuck during the measurements, see Figure 1. All indentations were carried out at 22 °C laboratory room temperature. Several load-controlled measurements were performed on the samples with different maximum loading forces to determine the most suitable value for comparison between the samples. Analyses of these different loading curves and the hardness and modulus results showed that, at very low loads, the measurements were slightly affected by instrumental noise (~1 mN), surface roughness, and tip–surface interaction uncertainties. At higher loads, substrate influence from the silicon wafer became progressively more pronounced. The raw varying maximum load measurements can be found in Supplementary File S1. The 10 mN maximum load was therefore selected as an optimal compromise, where the measurements exhibited stable hardness and modulus values, minimal noise, and penetration depths well below the commonly accepted 10% film-thickness criterion, thereby minimizing substrate effects. Static loading and analyses were performed according to the ISO 14577 standard [19]. A maximum loading force of 10 mN was applied at 60 different locations on the samples, with distances of 50 μm between each indent. The precise translations between the indentations were performed with the built-in computer-controlled motorized stage. The holding phase was set to 1 s (see Figure 2 for an example measurement). The instrument executed the loading and unloading segments in 20 discrete steps each, and at every step, the applied force and resulting displacement were recorded. The raw static measurements can be found in Supplementary File S1. A new Berkovich diamond tip, provided by Semilab, was used for the measurements, and the area function calibration was performed on fused silica; the compliance of the system was measured to be 0.3 nm/mN. The Poisson’s ratio of the SU-8 was taken as µ = 0.27 for the analysis. These measurements were used for hardness and elastic modulus determination using the Oliver–Pharr method [15].

### 2.3. Creep Nanoindentation Protocol

**Figure 3:**
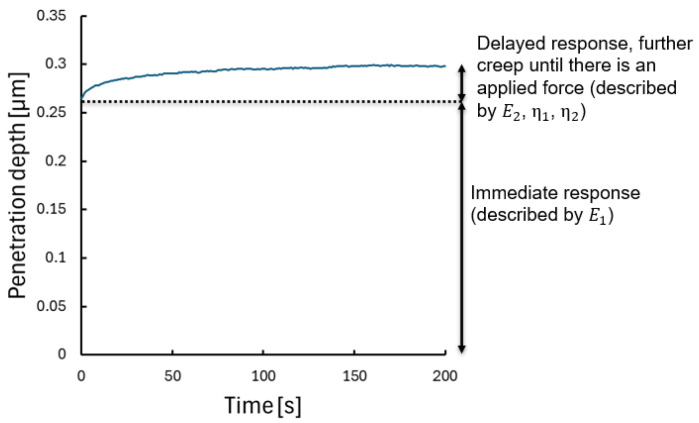
An example creep measurement curve: the immediate response of the material to the load at 0 s can be seen, while during the constant loading force, there is a delayed response.

To investigate the viscoelastic behaviour, creep measurements were performed with a Berkovich tip at 10 mN of constant loading force over 200 s (see Figure 3 for an example measurement). The displacement of the tip was continuously measured to observe the time-dependent deformation. The raw creep measurements can be found in Supplementary File S1. For the evaluation, the Burgers viscoelastic model was used, and it contains two ideally elastic springs ($E_{1}$ and $E_{2}$) and two ideal Newtonian dashpots ($\eta_{1}$ and $\eta_{2}$).

The selection of the Burgers viscoelastic model was based on a comparative evaluation of commonly used rheological representations for creep deformation. The Burgers model was selected because it simultaneously describes the instantaneous elastic response through a Hooke body ($E_{1}$, ideally elastic), the time-dependent recoverable deformation via a Kelvin–Voigt element ($E_{2},\;\;\eta_{2}$), and the irreversible viscous flow through a Newton body ($\eta_{1}$, ideally viscous). This combination enables the accurate representation of both short-term and long-term viscoelastic behaviour using a limited number of physically interpretable parameters. The Burgers model provided robust fitting quality across all baking phases while maintaining direct comparability with prior nanoindentation-based viscoelastic studies.

In the model, the immediate material response to the load is described by the $E_{1}$ modulus, while the long-term (time-dependent) creep features are governed by the $E_{2}$ retarded modulus and $\eta_{1}$ and $\eta_{2}$ viscosity values. The analytical solution to the time-dependent indentation depth *h*(*t*) is given by the following formula [20]:

(1) $$h^{2}\left(t\right)=\frac{\pi }{2}F_{0}\cot\left(\alpha \right)\left[\frac{1}{E_{1}}+\frac{1}{E_{2}}\left(1-e^{-t\frac{E_{2}}{\eta_{2}}}\right)+\frac{t}{\eta_{1}}\right]$$

### 2.4. Surface Morphology and Characterization

To investigate the morphology of indentation imprints on the SU-8 films, high-resolution images were captured via scanning electron microscopy (SEM) (Hitachi S-4700, Hitachi Scientific Ltd., Tokyo, Japan). High-resolution secondary electron images were captured at an acceleration voltage of 10 kV. The vacuum at the field-emission cathode and the sample was 10^−8^ Pa and 10^−6^ Pa, respectively. Prior to SEM imaging, the samples were coated with a 5–10 nm thick gold layer (Quorum Q150R Plus sputter coater) to eliminate surface charges and enhance image quality. SEM images can be found in Supplementary File S1.

### 2.5. Statistical Evaluation

For statistical evaluations, IBM SPSS statistical software (Version 23.0; IBM Corp., Armonk, NY, USA) was used. The mean moduli of elasticity and hardness were determined according to the Oliver–Pharr method, and samples were compared with an analysis of variance (ANOVA) test. Furthermore, a Bonferroni post hoc test was performed to compare the sample’s mean values. The significance level was set to 0.05. In the case of the creep test on each measured dataset, the least-squares fitting method was used to obtain the viscoelastic parameters. The results are reported as mean ± standard deviation.

## 3. Results

### 3.1. Static Load Test Results

**Figure 4:**
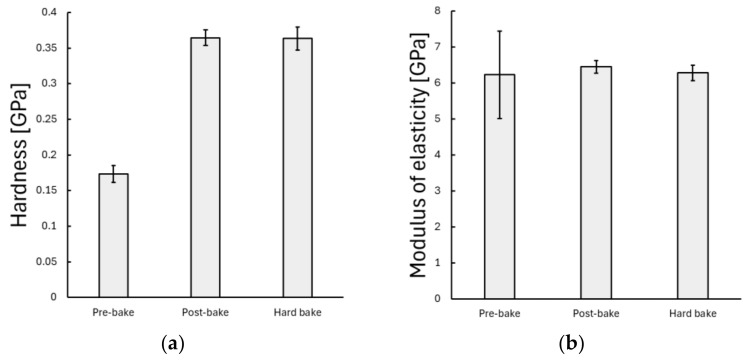
(**a**) Hardness and (**b**) modulus of elasticity results of the static load tests. The error bar shows the standard deviation.

**Figure A1:**
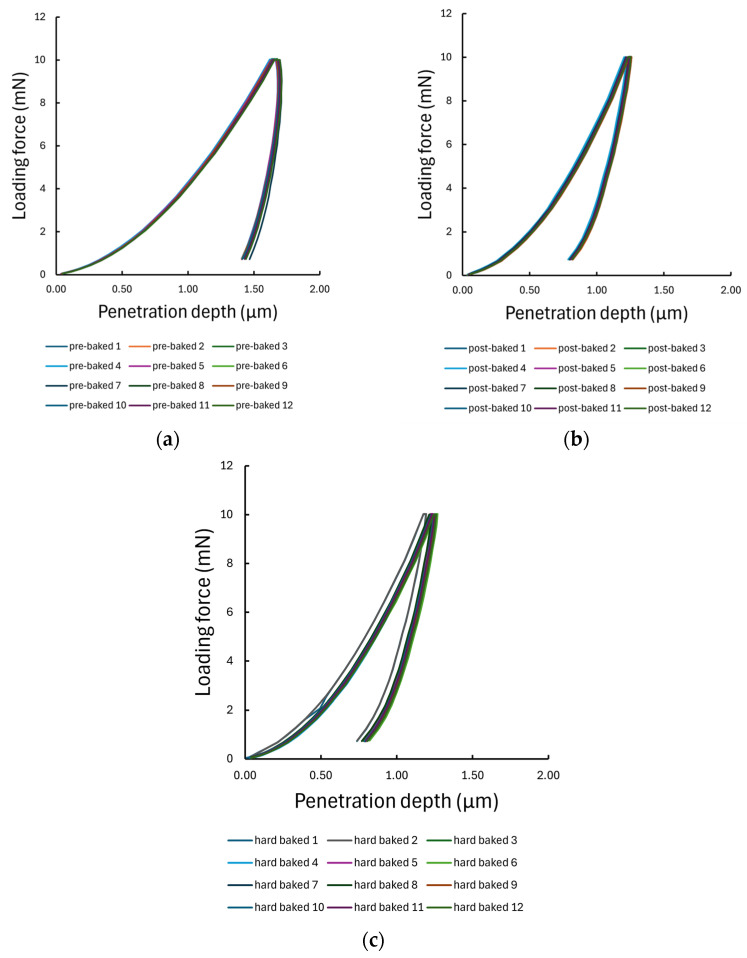
Nanoindentation measurements of the SU-8-coated silicon wafer at different baking phases. (**a**) shows the measured load-displacement curves of the pre-baked sample, (**b**) the post-baked and (**c**) the hard baked sample.

Nanoindentation experiments revealed significant differences in the mechanical properties of the SU-8-coated silicon wafer during different baking phases. The first 12 measurement results for each baking phase can be seen in Figure A1 in the Appendix A. After the pre-bake phase, the mean hardness of 0.173 ± 0.012 GPa (mean ± standard deviation) increased to 0.365 ± 0.011 GPa at the post-bake phase, and during the hard-bake phase, it remained at 0.364 ± 0.016 GPa (see Figure 4). The ANOVA test showed a significant difference between the mean hardness values $(p=8.2\times 10^{-142}$, ~26σ), which suggests a clear difference between the pre–post-bake and pre–hard-bake samples. This statistically significant difference was not observable in the case of the modulus of elasticity (stiffness of the coated layer, p=0.236). The mean modulus of elasticity values were very close to each other during different baking phases: 6.23 ± 1.21 GPa, 6.45 ± 0.17 GPa, and 6.28 ± 0.22 GPa in the order of pre-, post-, and hard-baked samples (see Figure 4). Interestingly, the pre-baked sample showed a higher deviation in the measured modulus values.

**Figure 5:**
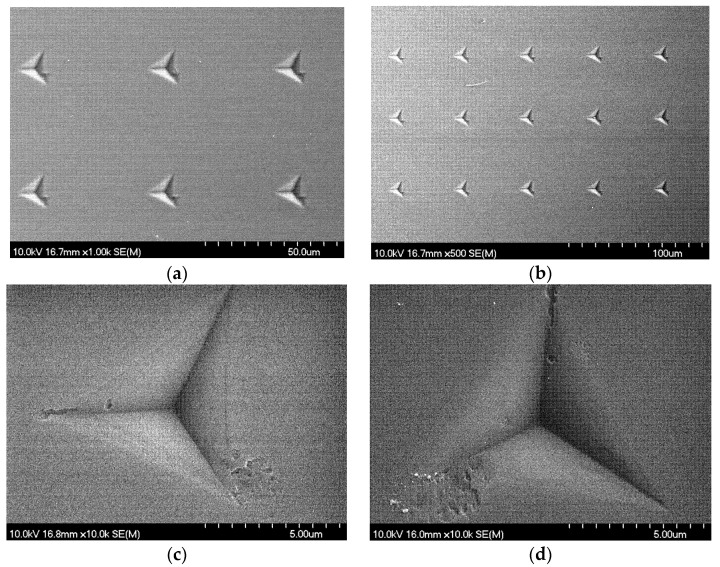
SEM images of the SU-8-coated wafer surfaces. (**a**,**b**) Images show arrays of nanoindentation imprints on the pre-bake sample with 50 µm spacing. (**c**,**d**) Images present high-magnification views of individual indents on the post-baked and hard-baked samples, respectively, revealing interfacial slipping around the imprint.

Berkovich imprints on the SU-8-coated wafers can be seen in Figure 5. The side lengths of the triangular indentations are approximately 5 µm. The edges and vertices are sharply defined, indicating consistent deformation. However, interestingly, localized cracking and slipping were observed along the edge of several indentations, suggesting that the mechanical response of the SU-8 coating can involve interfacial shear or brittle fracture under certain loading conditions. The overall adhesion and coating integrity remain high.

### 3.2. Creep Test Results

The evaluation of the Burgers model parameters revealed significant differences between different baking phases.

**Figure 6:**
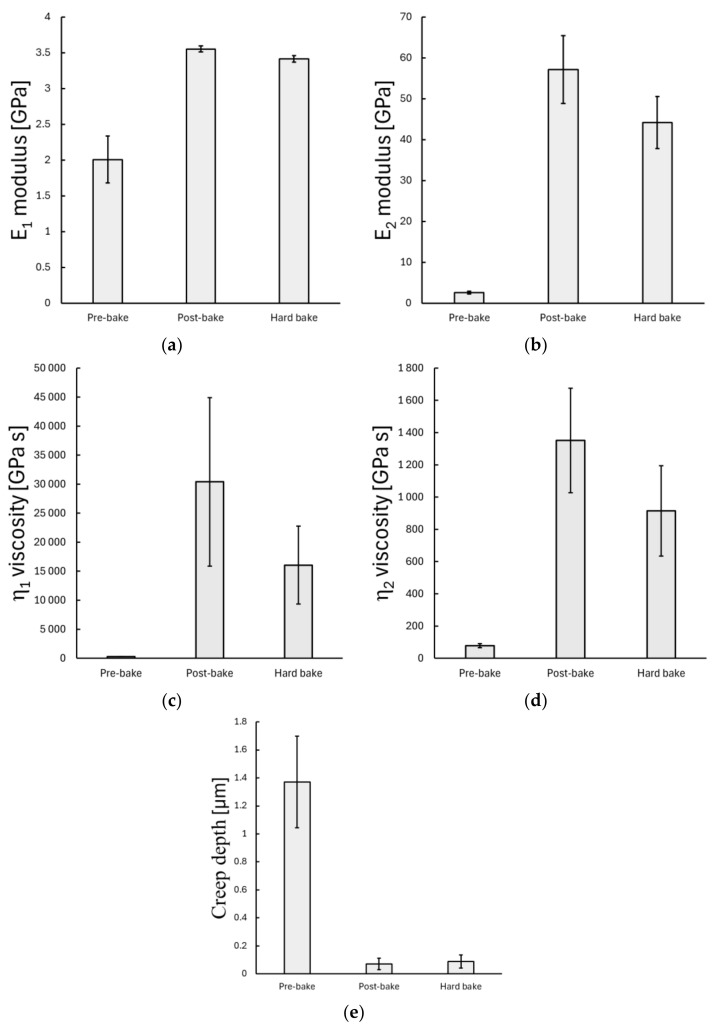
Viscoelastic parameters of SU-8-coated silicon wafer at different baking phases obtained by the creep nanoindentation method. (**a**–**d**) present the fitted viscoelastic parameters derived from the Burgers model, including the elastic modulus ($E_{1}$), retarded elastic modulus ($E_{2}$), and the corresponding viscosities ($\eta_{1}$ and $\eta_{2}$). The creep depth in Figure (**e**) represents the time-dependent displacement of the indenter during the creep phase following initial loading.

The instantaneous elastic modulus ($E_{1}$) increased significantly from 2.01 ± 0.33 GPa in the pre-baked phase to a mean value of 3.55 ± 0.04 GPa after the post-bake step (*p* < 0.001*). The hard-bake phase did not further increase the mean modulus value, where the mean modulus remained at 3.41 ± 0.46 GPa (*p* = 0.314) (see Figure 6a).

The retarded elastic modulus ($E_{2}$), which characterizes the time-dependent elastic feature, showed a significant difference among all baking phases. The pre-baked samples exhibited a mean retarded modulus of 2.6 ± 0.34 GPa, while the post-baked and hard-baked samples had statistically significantly higher mean values of 57.15 ± 8.29 GPa (*p* < 0.001 *) and 44.22 ± 6.36 GPa (*p* < 0.001 *), respectively (see Figure 6b).

The viscosity parameter $\eta_{1}$, representing linear time-dependent behaviour, was considerably lower in the pre-baked sample, with a mean value of 286 ± 32 GPa·s, and increased significantly after the post-bake step to 30 402 ± 14 514 GPa·s (*p* < 0.001 *). Following the hard-bake phase, the mean value decreased to 16 048 ± 6 705 GPa·s (*p* < 0.001*) (see Figure 6c).

A similar trend was observed for the viscosity parameter $\eta_{2}$, which describes the exponential time-dependent viscoelastic behaviour. The mean $\eta_{2}$ value increased from 77.71 ± 12.7 GPa·s in the pre-baked samples to 1351 ± 323 GPa·s (*p* < 0.001 *) after post-baking; then, it decreased to a mean value of 914 ± 280 GPa·s in the hard-baked sample (*p* < 0.001 *) (see Figure 6d).

The creep depth, reflecting the time-dependent displacement during the creep measurement after initial loading, was substantially greater in the pre-baked sample than in both post- and hard-baked samples. The mean creep displacement was 1.37 ± 0.083 µm for the pre-baked sample, whereas the post-baked and hard-baked samples showed only 0.071 ± 0.017 µm (*p* < 0.001 *) and 0.088 ± 0.012 µm (*p* < 0.001 *), respectively. No statistically significant differences were observed between the post- and hard-baked samples (*p* = 1.000).

## 4. Discussion

The nanoindentation experiments revealed significant changes in the hardness of the SU-8-coated silicon wafer samples associated with the baking phase, indicating progressive polymer cross-linking and solvent evaporation throughout the thermal processing steps. The measurements showed that the SU-8 coating exhibits a relatively high elastic modulus and hardness after the post-baking and hard-baking phases, confirming its suitability as a mechanically robust polymer layer for microfabricated structures. The values obtained by the Oliver–Pharr method showed good consistency across measurement points, indicating uniform coating quality. Post-bake and hard-bake measurements showed a statistically significant increase in the mean hardness values compared to the pre-baked phase, while the modulus of elasticity, describing the stiffness of the layer, did not change during static measurements. Creep experiments demonstrated a distinct time-dependent deformation component, which was well described by the Burgers viscoelastic model. The extracted parameters ($E_{1}$, $E_{2}$, $\eta_{1}$, and $\eta_{2}$) reflected the combined elastic and viscous behaviour of the SU-8 layer, with the instantaneous modulus dominating the short-term response and the retarded modulus governing long-term deformation. The measured creep depth values correlated with the viscous terms, supporting the validity of the model for polymeric coatings. The $E_{1}$ instantaneous modulus showed a significant increase after the post-bake and hard-bake phases compared to the pre-bake phase, similarly to the hardness results. However, the $E_{2}$, $\eta_{1}$, and $\eta_{2}$ time-dependant parameters showed a significant difference between all baking phases, describing different behaviours after these manufacturing processes. Overall, the results highlight the dual nature of SU-8 as a stiff yet viscoelastic material, emphasizing the importance of both elastic and time-dependent characterization in its mechanical assessment. The suitability of the Burgers model for this analysis is further supported by its ability to distinguish between instantaneous elasticity, retarded viscoelastic recovery, and long-term viscous flow, which evolve differently across the lithographic baking stages and cannot be resolved using simpler viscoelastic representations.

The static nanoindentation results obtained in this study are consistent with the literature values for SU-8 films fabricated on silicon substrates. Hwang et al. [21] reported modulus of elasticity values between 1.8 and 3.5 GPa from microbeam bending tests, emphasizing the influence of interfacial defects on apparent stiffness. In their study, they fabricated SU-8 layers as microcantilever beams on both polished and unpolished wafer surfaces, and the modulus increased with an increasing beam width, improving the surface’s quality. Their results highlighted the sensitivity of the measured modulus to fabrication-related defects and interfacial integrity, which is comparable to the slight variability observed in our static indentation data between differently baked samples. Our higher mean modulus of ~6 GPa agrees with later indentation-based studies, which are less affected by interfacial compliance and probe the near-surface region of fully cured films. Similar magnitudes of modulus values were reported by Krishna et al. [22], who obtained 6.5 ± 0.05 GPa for the modulus and 455 MPa for hardness using quasi-static indentation without significant size or rate effects, confirming that this range represents the intrinsic stiffness of cross-linked SU-8. Their study demonstrated that the modulus does not depend on the loading rate and holding time. Nanoindentation measurements predominantly sense the near-surface, fully polymerized region of the coating and reflect the higher degree of cross-linking achieved under current thermal processing parameters.

When nanoindentation is performed on thin films supported by stiff substrates, the contribution of the substrate to the measured mechanical response must be carefully considered. It is well established that substrate effects become significant when the indentation penetration depth exceeds approximately 10% of the film thickness, potentially leading to an overestimation of both elastic modulus and hardness for compliant coatings on rigid substrates [20,23]. In our case, the SU-8 film’s thickness was 22–25 μm, while the maximum indentation depth at the applied peak load of 10 mN was approximately 1.3 μm. This corresponds to a penetration depth-to-thickness ratio of about 5–6%, which is well below the commonly accepted threshold for the onset of significant substrate influence. The mechanical response measured under these conditions can be considered to predominantly reflect the intrinsic near-surface properties of the SU-8 coating rather than the underlying silicon wafer. This is also supported by the experimental observation that the measured elastic modulus values (~6 GPa) are in close agreement with the previously reported nanoindentation-based measurements of fully cured SU-8 films [22] while remaining orders of magnitude lower than the elastic modulus of the silicon substrate. Taken together, these considerations indicate that substrate effects are negligible under the applied experimental conditions, and the reported elastic, plastic, and viscoelastic parameters represent a valid approximation of the intrinsic mechanical behaviour of the SU-8 coating in the near-surface region.

The influence of processing and structural anisotropy on the mechanical performance of SU-8 has been highlighted in recent microtensile studies. Robin et al. [24] examined 2 μm thick spin-coated SU-8 2002 films using in situ optical tensile experiments combined with digital image correlation to quantify full-field strain distributions. They reported mean modulus values of 3.48 ± 0.57 GPa for hard-baked and 2.92 ± 0.43 GPa for non-hard-baked films, accompanied by a ≈20% increase in modulus and strength after hard baking due to enhanced cross-linking, as confirmed by FTIR. Furthermore, the authors demonstrated that spin-coating induces in-plane anisotropy through radial shear flow, leading to direction-dependent tensile moduli and strength. Their analysis indicated that modulus values obtained from nanoindentation tests are typically 1.5–2× higher than those from tensile loading because of out-of-plane anisotropy and substrate constraint. These findings agree with our observation that the localized indentation of fully cross-linked near-surface regions yields higher apparent moduli (~6 GPa), whereas in-plane mechanical measurements or bulk bending tests yield lower values. Micromechanical investigations by Cherukuri et al. [25] were used to conduct in situ SEM micropillar compression and Berkovich nanoindentation experiments on photolithographically patterned SU-8 films across seven orders of magnitude in strain rate (10^−3^–10^3^ s^−1^), which also agrees with our quasi-static results. They reported that the elastic modulus remains nearly constant at ~4–5 GPa irrespective of the strain rate, while hardness and yield stress increase modestly. Their findings confirm that SU-8 behaves as a rate-insensitive elastic solid but exhibits thermally activated plasticity at high deformation rates. This reinforces that the primary differences between reported values arise from process-dependent cross-linking density and measurement depth rather than from viscoelastic rate effects. Our post- and hard-baked samples show slightly higher moduli (~6 GPa), which is consistent with a denser polymer network produced by extended thermal curing.

Temperature-controlled indentation by Chang et al. [26] showed a moderate softening between 25 °C and 45 °C of the SU-8 using a Berkovich indenter integrated with an AFM-based nanoindenter system. They found a clear thermal softening trend, where the modulus decreased from approximately 14 GPa at 25 °C to 11 GPa at 45 °C under a 1 mN load, while hardness dropped from 0.43 GPa to 0.26 GPa. The authors attributed this decline to the temperature-induced mobility of the epoxy network, reducing effective cross-link density. In addition, they observed that apparent modulus and hardness increase with indentation load, an effect primarily caused by the stiffer silicon substrate contribution. These findings underline the pronounced temperature sensitivity of SU-8 and emphasize that the near-surface mechanical response depends not only on baking parameters but also on the thermal environment during testing and operation. In the present study, all measurements were performed at room temperature under quasi-static loading; thus, the reported moduli around 6 GPa reflect the intrinsic stiffness of the SU-8 coating in the fully cured state, without significant substrate or temperature-related deviations.

The time- and temperature-dependent deformation behaviour obtained from creep measurements agrees with previous viscoelastic characterizations of SU-8. Pustan et al. [27] systematically investigated the evolution of the elastic modulus of SU-8, along with hardness, adhesion, and friction, using AFM-based nanoindentation and spectroscopy at operating temperatures between 20 °C and 100 °C for samples hard-baked at 125–215 °C. Their results revealed a marked softening with increasing temperature: The elastic modulus decreased by 47–53%, and hardness decreased by 46–70%, whereas adhesion and friction forces increased by up to 40% and 80%, respectively. These changes were attributed to thermal relaxation and enhanced chain mobility in the polymer network, consistent with the onset of viscoelastic flow well below the glass-transition temperature ($T_{g}$ ~ 210 °C). The viscoelastic nature of SU-8 has also been studied by Chung and Park [28], who combined tensile and dynamic mechanical analysis (DMA) to study its temperature- and frequency-dependent response between 25 °C and 200 °C. Their results revealed that the modulus decreases steadily with temperature, falling by nearly 60% as the polymer approaches its glass-transition region. DMA measurements showed characteristic peaks in the loss modulus and loss factor (tan δ) between 125 °C and 150 °C, marking the transition from glassy to rubbery behaviour. Increasing the excitation frequency (0.1–10 Hz) shifted this transition toward higher temperatures, reflecting thermally activated segmental mobility typical of cross-linked epoxies. Importantly, prolonged thermal exposure (3 h at 150 °C) led to the stabilization of the storage modulus and a right-shift of the loss factor maximum, indicating post-curing and increased cross-link density. These finding agrees with our Burgers model analysis, where the retarded modulus $E_{2}$ and viscosity $\eta_{2}$ showed significant increases after the post-bake step, suggesting a similar reduction in molecular relaxation pathways. Together, these results confirm that the time- and temperature-dependent deformation observed in SU-8 arises from a combination of thermally driven chain relaxation and curing-related stiffening, consistent with the viscoelastic behaviour of other highly cross-linked epoxy systems.

The viscoelastic interpretation of our creep data is also strongly supported by the microscale analysis of Xu et al. [11], who combined micropillar compression and viscoelastic nanoindentation of SU-8 2025 films fabricated by UV lithography. Their study demonstrated that SU-8 exhibits measurable viscoelastic relaxation even at room temperature, with the mean modulus increasing from ~3.1 to 4.8 GPa as the strain rate rose from 10^−4^ to 10^−2^ s^−1^. When the same material was analysed using the classical Oliver–Pharr approach, the apparent modulus was ~6 GPa, an overestimation caused by neglecting viscoelastic compliance during unloading. By applying Lu’s nanoindentation-based viscoelastic analysis, Xu et al. extracted relaxation and creep parameters that closely matched the tensile test results reported by Robin et al. [24]. Their findings confirm that the instantaneous stiffness of SU-8 is higher than its long-term equilibrium modulus and that the measured parameters depend on the strain-rate window and load-hold protocol. This behaviour is consistent with our Burgers model analysis, in which the retarded modulus $E_{2}$ and viscosities $\eta^{1}\;and\;\eta_{2}$ capture the same time-dependent stress-relaxation effects observed by Xu et al. The studies of Schiffmann and Brill [29] complement these results. They systematically investigated SU-8 thin films at each stage of the lithographic process using nanoindentation-based creep and stress-relaxation tests. By deriving time-dependent viscoelastic functions, they quantified how cross-linking progressively transforms SU-8 from a viscous liquid-like resist into a predominantly elastic solid. The dynamic viscosity increased by nearly fifty-fold during cross-linking, and the retardation spectra revealed a strong reduction in long relaxation times as curing advanced. Notably, the fully processed SU-8 films exhibited ~75% total elastic recovery (immediate + retarded) after three days, while pre-baked films only showed ~25%, demonstrating the transition from viscous to viscoelastic-elastic behaviour. These results are fully consistent with our Burgers model analysis, where the post-bake step caused marked increases in the retarded modulus ($E_{2}$) and viscosities ($\eta_{1}$ and $\eta_{2}$), accompanied by a reduction in long-time creep. Collectively, these experimental and modelling observations confirm that post-bake processing increases cross-link density and suppresses viscoelastic flow, stabilizing the mechanical response of SU-8 across thermal and temporal scales. Despite this general consistency with earlier viscoelastic investigations, an important distinction must be emphasized. Previous nanoindentation-based creep and stress-relaxation studies of SU-8 primarily addressed either broad curing trends, temperature-dependent behaviour, or generic stages of polymerization without direct alignment to the discrete baking steps used in standard photolithographic processing. In contrast, the present work explicitly maps the evolution of Burgers model parameters onto the pre-bake, post-exposure-bake, and hard-bake phases applied to wafer-supported SU-8 coatings. This process-resolved approach reveals that the most pronounced changes in viscoelastic response occur during the post-bake step, where the retarded modulus and viscosity increase dramatically, while further hard-baking mainly stabilizes these parameters. Such differentiation cannot be inferred from static indentation alone and provides direct mechanistic insight into how thermal curing suppresses long-term viscoelastic deformation through increasing cross-link density.

The strong dependence of both elastic and viscoelastic properties on the thermal history of SU-8 underscores the importance of optimized curing conditions in MEMS and microfluidic applications. A sufficiently long post-bake or hard-bake step ensures stable modulus and minimized creep under mechanical or thermal loading. Because nanoindentation directly captures both the instantaneous and time-dependent responses, it provides a rapid, nondestructive means of assessing polymerization completeness and uniformity across wafers. The present results, therefore, not only clarify the mechanical evolution of SU-8 during processing but also support its use as a process-monitoring and quality-control tool for polymer-based microsystems.

## 5. Conclusions

This study systematically characterized the mechanical behaviour of SU-8 thin coats on silicon wafers through static and time-dependent nanoindentation at different baking stages. The results demonstrate a strong correlation between the degree of thermal curing and both the instantaneous and viscoelastic properties of the polymer. The post-bake process produced a marked increase in hardness and elastic modulus, while the creep and relaxation tests revealed a concurrent rise in the retarded modulus and viscosities of the Burgers model, indicating a substantial reduction in long-term viscoelastic flow.

The mechanical behaviour of SU-8 is primarily governed by the extent of cross-linking rather than by strain rate or size effects. The increase in modulus and viscosity with baking temperature reflects the transition from a partially polymerized resist to a highly cross-linked, glassy network.

These findings underline the importance of optimized post-bake or hard-bake conditions to achieve mechanically stable SU-8 layers for MEMS and microfluidic applications. Furthermore, the nanoindentation-based evaluation of both instantaneous and creep responses provides a rapid and nondestructive method for monitoring polymerization completeness and coating uniformity, offering a practical route for the process control and mechanical qualification of SU-8 coatings in microfabrication.

## Data Availability

The original contributions presented in this study are included in the article/Supplementary Material. Further inquiries can be directed to the corresponding author.

## Supplementary Materials

The following supporting information can be downloaded at https://www.mdpi.com/article/10.3390/polym17243337/s1. The Supplementary File contains raw data of the measurements used for the analyses presented in this study, along with the SEM images and premeasurements with variable loading from 1 mN to 400 mN.

## Appendix A

The first 12 representative load–displacement curves for all three groups are provided in the Appendix A (see Figure A1) to illustrate the nanoindentation behaviour. In the case of the pre-baked sample, the displacement at the maximum of 10 mN loading reaches slightly higher than 1.5 µm, while in the post- and hard-bake cases, it is around 1.25 µm. The variations in the curves are low in all three cases.

## References

[1] Das A., Sinha A., Rao V.R., Jonnalagadda K.N. (2017). Fracture in Microscale SU-8 Polymer Thin Films. Exp. Mech..

[2] Jiguet S., Bertsch A., Judelewicz M., Hofmann H., Renaud P. (2006). SU-8 Nanocomposite Photoresist with Low Stress Properties for Microfabrication Applications. Microelectron. Eng..

[3] Feng R., Farris R.J. (2002). The Characterization of Thermal and Elastic Constants for an Epoxy Photoresist SU8 Coating. J. Mater. Sci..

[4] Lorenz H., Despont M., Fahrni N., LaBianca N., Renaud P., Vettiger P. (1997). SU-8: A Low-Cost Negative Resist for MEMS. J. Micromech. Microeng..

[5] del Campo A., Greiner C. (2007). SU-8: A Photoresist for High-Aspect-Ratio and 3D Submicron Lithography. J. Micromech. Microeng..

[6] Lee J., Choi K.-H., Yoo K. (2014). Innovative SU-8 Lithography Techniques and Their Applications. Micromachines.

[7] Feng R., Farris R.J. (2002). Influence of Processing Conditions on the Thermal and Mechanical Properties of SU8 Negative Photoresist Coatings. J. Micromech. Microeng..

[8] Butikova J., Pervenecka J., Vitols K., Tropins E., Vanags E., Bundulis A., Einbergs E., Vembris A., Grube J. (2024). Exposure and Post-Bake Thermal Treatment in One Step for SU8 Photoresist. Nano-Struct. Nano-Objects.

[9] Matarèse B.F.E., Feyen P.L.C., Falco A., Benfenati F., Lugli P., deMello J.C. (2018). Use of SU8 as a Stable and Biocompatible Adhesion Layer for Gold Bioelectrodes. Sci. Rep..

[10] Zhang J. (2016). Lattice Boltzmann Method and Its Applications in Microfluidics. Microfluidics and Nanofluidics Handbook.

[11] Xu T., Yoo J.H., Babu S., Roy S., Lee J.-B., Lu H. (2016). Characterization of the Mechanical Behavior of SU-8 at Microscale by Viscoelastic Analysis. J. Micromech. Microeng..

[12] Williams J.D. (2004). Study on the Postbaking Process and the Effects on UV Lithography of High Aspect Ratio SU-8 Microstructures. J. Micro/Nanolith. MEMS MOEMS.

[13] Anhoj T.A., Jorgensen A.M., Zauner D.A., Hübner J. (2006). The Effect of Soft Bake Temperature on the Polymerization of SU-8 Photoresist. J. Micromech. Microeng..

[14] Keller S., Blagoi G., Lillemose M., Haefliger D., Boisen A. (2008). Processing of Thin SU-8 Films. J. Micromech. Microeng..

[15] Oliver W.C., Pharr G.M. (1992). An improved technique for determining hardness and elastic modulus using load and displacement sensing indentation experiments. J. Mater. Res..

[16] Li W., Zhao X., Xie G., Luo X., Cheng X., Su Y. (2025). Programmable High-Performance Ternary Piezoelectric Nanogenerators by Synergizing Reinforcement Effect and Percolation Effect. Mater. Today Chem..

[17] Dai J., Li H., Que L., Xie G., Su Y. (2025). Wide-Range Human Physiological Signal Acquisition with Carbonized Composite Nanofibers. Nanoscale.

[18] Liu C.-K., Lee S., Sung L.-P., Nguyen T. (2006). Load-Displacement Relations for Nanoindentation of Viscoelastic Materials. J. Appl. Phys..

[19] (2015). Metallic Materials—Instrumented Indentation Test for Hardness and Materials Parameters—Part 1: Test Method.

[20] Fischer-Cripps A.C. (2011). Nanoindentation.

[21] Hwang S.-F., Yu J.-H., Lai B.-J., Liu H.-K. (2008). Young’s Modulus and Interlaminar Fracture Toughness of SU-8 Film on Silicon Wafer. Mech. Mater..

[22] Krishna B., Singh R.K., Mishra Y.K. (2025). Nanomechanical Properties of SU8-Photoresist Thin Films. J. Elastomers Plast..

[23] Fischer-Cripps A.C. (2007). Introduction to Contact Mechanics.

[24] Robin C.J., Vishnoi A., Jonnalagadda K.N. (2014). Mechanical Behavior and Anisotropy of Spin-Coated SU-8 Thin Films for MEMS. J. Microelectromech. Syst..

[25] Cherukuri R., Lambai A., Sukki L., Väliaho J., Kallio P., Sarlin E., Ramachandramoorthy R., Kanerva M., Mohanty G. (2024). In-Situ SEM Micropillar Compression and Nanoindentation Testing of SU-8 Polymer up to 1000 S−1 Strain Rate. Mater. Lett..

[26] Chang R.-C., Chen F.-Y., Yang P.-H. (2006). Mechanical properties of photoresist thin films at various temperature. J. Chin. Soc. Mech. Eng..

[27] Pustan M., Birleanu C., Voicu R., Muller R. (2022). AFM Characterization of Temperature Effect on the SU-8 Mechanical and Tribological Properties. Polymers.

[28] Chung S., Park S. (2013). Effects of Temperature on Mechanical Properties of SU-8 Photoresist Material. J. Mech. Sci. Technol..

[29] Schiffmann K.I., Brill C. (2007). Testing the Viscoelastic Properties of SU8 Photo Resist Thin Films at Different Stages of Processing by Nanoindentation Creep and Stress Relaxation. Int. J. Mater. Res..

