# Supplementary figures and images for: Nanoindentation Analysis of SU-8 Coated Wafers at Different Baking Phases
Source: Polymers (Basel). 2025 Dec 18;17(24):3337. doi: 10.3390/polym17243337 (PMC12736521; doi:10.3390/polym17243337)

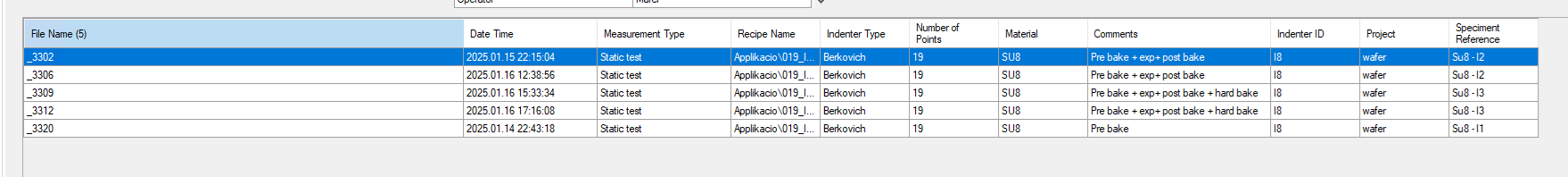

Supplement: Supplementary file 1 [file polymers-17-03337-s001.zip › supplementary/pre measurements with variable loadings/high.png]

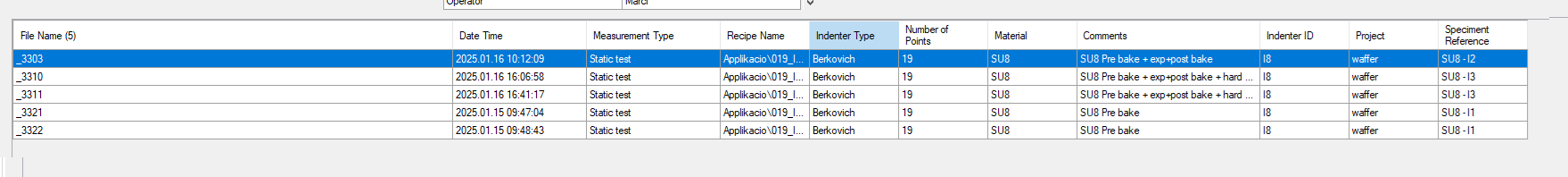

Supplement: Supplementary file 1 [file polymers-17-03337-s001.zip › supplementary/pre measurements with variable loadings/med.png]

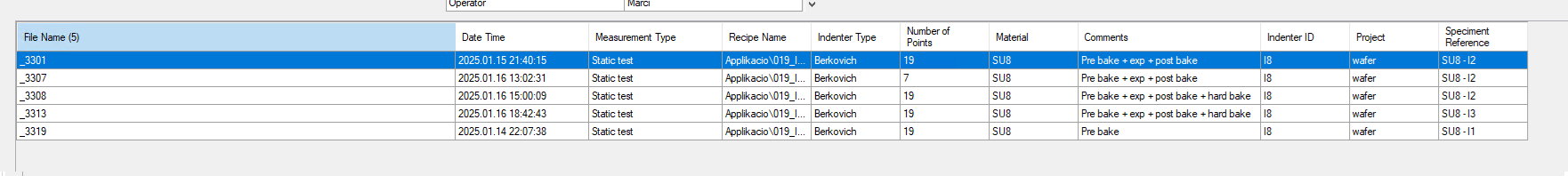

Supplement: Supplementary file 1 [file polymers-17-03337-s001.zip › supplementary/pre measurements with variable loadings/vhigh.png]
